# Blood purification in sepsis

**DOI:** 10.1186/s13054-018-2286-4

**Published:** 2018-12-22

**Authors:** Achim Jörres

**Affiliations:** Department of Medicine I - Nephrology, Transplantation & Medical Intensive Care, University Witten/Herdecke, Medical Center Cologne-Merheim, Ostmerheimer Str. 200, D-51109 Cologne, Germany

**Keywords:** Sepsis, Blood purification, Hemoperfusion, Therapeutic plasma exchange

## Introduction

The mortality of patients with severe sepsis and septic shock remains unacceptably high. Thus, there is an urgent clinical need for novel therapeutic approaches to improve the prognosis of these patients. At present, apart from antibiotic therapy and infectious source control, the mainstay of therapy is symptomatic. However, research has led to a better understanding of the pathophysiology of sepsis, in which the activation of multiple pro- and anti-inflammatory mediators plays a key role [1]. Whilst animal models of sepsis have provided encouraging results with strategies aiming at modulation of these pathways, clinical studies in patients using targeted pharmacological approaches have so far proved disappointing.

Recently, however, attempts to improve the outcome of sepsis patients by extracorporeal immunomodulation have seen a certain renaissance, with novel or not-so-novel devices, such as CytoSorb cytokine hemoadsorption and polymyxin B (Toraymyxin) endotoxin adsorption, being studied in multicenter randomized clinical trials (RCTs).

Toraymyxin is an extracorporeal hemoperfusion device employing immobilized polymyxin B (PMX) to remove circulating endotoxin by adsorption. Developed in Japan in the early 1990s, a first European multicenter pilot trial in 36 surgical patients with severe sepsis or septic shock secondary to intraabdominal infection demonstrated that the treatment is safe and may lead to improvement in renal and cardiac parameters [2]. Another multicenter RCT in Italy studied 64 patients with severe sepsis/septic shock from intra-abdominal Gram-negative infections and reported that PMX hemoperfusion significantly improved hemodynamics and organ dysfunction and reduced 28-day mortality [3]. However, two subsequent larger clinical studies were negative. A French multicenter RCT included 243 patients with septic shock after emergency abdominal surgery who either received two hemoadsorption sessions in addition to conventional therapy or conventional therapy alone. PMX therapy led to a non-significant increase in mortality and no improvement in organ failure [4]. The recent EUPHRATES trial in North America enrolled 450 adult critically ill patients with septic shock and an endotoxin activity assay level of ≥ 0.60 to receive two PMX treatments or sham hemoperfusion in addition to standard therapy. PMX hemoadsorption was not associated with a significant difference in mortality at 28 days among all participants nor in the population with greater severity of illness (MODS > 9) [5].

CytoSorb is a hemoadsorption device containing porous polymeric beads capable of removing cytokines and other middle-molecular weight compounds (up to 55 kDa) from blood by size exclusion and surface adsorption. It was recently studied in a multicenter RCT in 100 mechanically ventilated patients with severe sepsis or septic shock and acute lung injury or ARDS. Patients were randomly assigned to either therapy with CytoSorb hemoperfusion (for 6 h per day for up to 7 consecutive days) in addition to standard therapy or to standard medical therapy alone. Primary outcome was change in interleukin (IL)-6 serum concentrations. Whilst significant IL-6 elimination, averaging between 5 and 18% per blood pass throughout the entire treatment period, was found, this did not lead to lower plasma IL-6 levels. Moreover, in the unadjusted analysis 60-day mortality was significantly higher in the hemoperfusion group. After adjustment for patient morbidity and baseline imbalances, however, no association of CytoSorb hemoperfusion with mortality was found [6]. These results have clearly damped the enthusiasm that appeared to have grown following positive reports from case series and non-randomized studies with this form of therapy. Consequentially, in the absence of compelling clinical data, the present Surviving Sepsis Campaign guidelines do not provide a recommendation regarding the use of blood purification techniques in patients with sepsis [7].

Therapeutic plasma exchange (TPE) therapy may not only ameliorate peak concentrations of pro-inflammatory and antifibrinolytic molecules but also contribute to the restitution of a less hostile plasma milieu via infusion of “healthy” fresh frozen plasma containing, e.g., anti-coagulant proteins and ADAMTS13. At present, however, only limited clinical evidence is available in using TPE in patients with sepsis. The largest RCT to date randomized 106 patients with severe sepsis/septic shock to receive either standard therapy or additional plasma exchange and reported a lower 28-day mortality rate with TPE. However, when controlled for other contributing factors, the effect of TPE on mortality became a non-significant trend (*P* = 0.07) [8]. A systematic review and meta-analysis identified only four randomized clinical studies including a total of 194 patients, concluding that insufficient evidence exists to recommend TPE as an adjunctive therapy for patients with sepsis [9]. Correspondingly, in their “Guidelines on the use of therapeutic apheresis in clinical practice” the American Society for Apheresis (ASFA) places TPE only in their indication category III (“Optimum role of apheresis therapy is not established. Decision making should be individualized”) as regards the treatment of sepsis with multiorgan failure [10].

In this journal, Knaup and coworkers report on a prospective non-randomized pilot study of early therapeutic plasma exchange in 20 patients within 12 h of onset of septic shock and requiring high doses of norepinephrine [11]. TPE was well tolerated and resulted in rapid reduction of norepinephrine doses required to maintain MAP > 65 mmHg. Moreover, favorable changes in the cytokine profile were observed. Given the small patient number in this pilot study it obviously remains unknown whether early TPE also may improve survival and other clinical endpoints in these patients. This important issue will ultimately have to be clarified by a sufficiently powered, randomized prospective clinical trial. Knaup and coworkers must be lauded, however, for having demonstrated that such a trial, and with early intervention at that, is indeed feasible and potentially promising.

## Conclusions

The clinical evidence to date supporting extracorporeal blood purification for removal of endotoxins and/or proinflammatory mediators in sepsis is mostly limited to case series and non-randomized studies while the results from most RCTs have so far been disappointing. On the other hand, therapeutic plasma exchange might offer an additional benefit as it not only removes potential culprits from patients’ blood but may also contribute to the restoration of plasmatic homeostasis via infusion of healthy donor plasma. Recent data suggest that early TPE in sepsis is both safe and feasible. Its clinical efficacy, however, remains to be established by prospective clinical endpoint studies.

## Abbreviations

ADAMTS13: A disintegrin-like metalloproteinase with thrombospondin motif type 1 member 13
IL-6: Interleukin-6
PMX: Polymyxin B
RCT: Randomized controlled trial
TPE: Therapeutic plasma exchange
